# Mobile User Indoor-Outdoor Detection through Physical Daily Activities

**DOI:** 10.3390/s19030511

**Published:** 2019-01-26

**Authors:** Aghil Esmaeili Kelishomi, A.H.S. Garmabaki, Mahdi Bahaghighat, Jianmin Dong

**Affiliations:** 1 MOE Key Laboratory for Intelligent and Network Security, Xi’an Jiaotong University, Xi’an 710049, China; jianmind23@stu.xjtu.edu.cn; 2 Division of Operation and Maintenance Engineering, Luleå University of Technology, 97187 Luleå, Sweden; amir.garmabaki@ltu.se; 3 Department of Electrical Engineering, Raja University, 34148 Qazvin, Iran; m.bahaghighat@raja.ac.ir

**Keywords:** sensor-based indoor-outdoor detection, location-based services, human daily activity, smartphone motion sensors, machine learning, context awareness

## Abstract

An automatic, fast, and accurate switching method between Global Positioning System and indoor positioning systems is crucial to achieve current user positioning, which is essential information for a variety of services installed on smart devices, e.g., location-based services (LBS), healthcare monitoring components, and seamless indoor/outdoor navigation and localization (SNAL). In this study, we proposed an approach to accurately detect the indoor/outdoor environment according to six different daily activities of users including walk, skip, jog, stay, climbing stairs up and down. We select a number of features for each activity and then apply ensemble learning methods such as Random Forest, and AdaBoost to classify the environment types. Extensive model evaluations and feature analysis indicate that the system can achieve a high detection rate with good adaptation for environment recognition. Empirical evaluation of the proposed method has been verified on the HASC-2016 public dataset, and results show 99% accuracy to detect environment types. The proposed method relies only on the daily life activities data and does not need any external facilities such as the signal cell tower or Wi-Fi access points. This implies the applicability of the proposed method for the upper layer applications.

## 1. Introduction

One of the most significant technology trends in the current decade is an enormous proliferation of smart mobile devices in daily life. As reported in [1], the number of current smartphone users is 2.53 billion, with growth expected to 2.87 billion by 2020. The extensive usage of smartphones in society makes them an important platform that serves the ubiquitous sensing and communication needs of people [2]. The influence of smart mobile devices and increasing the ability to access the internet anywhere represent a high motivation for producing mobile applications based on location-based systems (LBSs). The LBS field plays a crucial role in many domains, including tracking, navigation, safety-related services, location-sensitive billing, advertising, tourism, healthcare monitoring, intelligent transportation, etc. [3,4,5]. Therefore, the LBS sector is receiving significant research attention from academia and industry. However, all mobile applications based on LBS have a common requirement: the *current user positioning*. Since mobile users can be in many places such as open sky outdoors, crowded avenues, indoor environments, etc., the next generation of positioning systems has to perform well both indoors and outdoors. The Global Positioning System (GPS) is good enough for outdoor environment positioning and recently some accurate indoor positioning systems (IPSs) have been developed using wireless networks [6], RFID [7], Wi-Fi [8,9], Bluetooth [10,11], and geomagnetism intensity [12]. If the IPS is successfully built and commercialized, then someday the integration of GPS and IPS will be a solution for the indoor-outdoor integrated positioning system. However, switching between GPS and IPS is still a challenge, where for the convenience of users the switching should occur automatically, fast, precisely and with efficient power consumption.

Generally, there are two categories of techniques for indoor-outdoor (IO) sensing methods: GPS-based and smartphone sensor-based. The GPS-based techniques use the degradation of the GPS signal when going from the outdoor to the indoor environment as the primary parameter to distinguish the user IO status. This degradation of the GPS signal happens when GPS satellite signals are absorbed or attenuated due to blocking walls or ceilings. The two potential issues of GPS-based IO detectors limit their capacity to determine the environment. First, in many cases there is not enough signal degradation that can help indicate the change in user status. For example, in [13,14] we are reminded that GPS signals are still able to demonstrate the user position in buildings with large windows, like in the outdoor environment, and it is not possible to conclude the user IO state confidently. In such cases, GPS signal cannot serve as a user IO state detector, but it can aid in user positioning. The second flaw is that GPS sensor is the most power-hungry sensor compared to other smartphone sensors, and it has been shown in [15,16] that utilizing GPS consumes seven-times more energy than the accelerometer and gyroscope sensors accessible on a smartphone. Furthermore, the GPS signal needs a period of 10 to 35 s to fix its state after moving between the outdoor and indoor environment [17].

The sensor-based methods utilize the other built-in sensors of the smartphone to detect the user IO state to overcome the GPS limitations and they can be used opportunistically to save power. These sensors are Wi-Fi, Bluetooth, ambient light, GSM, microphone, cell, accelerometer, magnetometer, and proximity. Since the intensity level of a user’s surrounding environment properties (i.e., light [18], magnetic field [19], temperature [20], sound [21], GSM signals [22], and Wi-Fi [23]) can be different when the user has a transition from indoors to outdoors or vice versa, these intensity differences are measured as the main parameters of the sensor-based methods to detect the user IO state. However, the light-based methods may not be able to obtain enough light intensity variance under some conditions, such as specific times of the day (e.g., at dawn and dusk) [24], or the way of the user interacting with a smartphone (e.g., placing the smartphone in a bag or pocket). The proximity sensor is utilized to assist the decision of light sensors when the light intensity change is due to an object blocking the light sensor or a changing environment. Since the intensity of the Bluetooth signal is scattered randomly and is reduced over distance [25,26], they are installed at the specific points of indoor environment entrances that measure the changing values of received signal strength (RSS) to indicate the user IO state. Considering the geomagnetic field has smooth attributes outdoors and can be disturbed by ferromagnetic materials such as iron, steel, and other similar structures indoors, a thresholding value of the geomagnetic field can be used for indoor-outdoor detection. The thresholding value is determined based on the calculated variance of the geomagnetic field for a number of frames. Cellular signals are another potential signal surrounding mobile users, which is provided by cell towers. The cellular signal strength suffers a substantial drop similar GPS signal conditions when the user moves from outdoors to indoors and vice versa, that the signal drop difference is used to identify the environment type. However, sometimes the prolonged transition between indoors and outdoors or the reverse can lead to a longer time for collecting data from the tower, which can increase the battery consumption. The same principle is used by Wi-Fi approaches to detect the user IO state, as the Wi-Fi access points (APs) must be scanned, which requires more time and energy consumption than other smartphone sensors.

Later on, multi-model approaches such as IODetector [18] and SenseIO [24] were introduced to overcome the above sensor issues by using different sensors as subdetectors to cover the weaknesses of single-approach methods. Despite their excellent accuracy, the multi-model approaches suffer power consumption drawbacks and from erroneous decisions because of their use of different sensors and connections between the previous and current state of the user to decide when to switch between two sensors.

These shortcomings of the abovementioned approaches are our motivation is for this work. Since daily human activities can be measured by the accelerometer and gyroscope sensors of the smartphone, we propose an accurate single-modal approach based on a user’s daily physical activities. The proposed method is independent of all other environmental facilities such as APs and cell towers or environmental conditions such as intensity of light, sound, and the magnetic field. In this study, only the primary daily activities collected by accelerometer and gyroscope sensor data are used as input to determine the user IO status. Furthermore, a comprehensive empirical model evaluation on the smartphone users’ daily activities to understand the effects of each activity on recognition accuracy rates and their robustness is performed. The performance of the model has been verified under several various changing factors such as the data fusion of motion sensors, the degree of imbalanced data and sensitivity to the size of the features’ dataset. In addition, we propose a comprehensive sensor-feature database of different operational properties including time-, frequency-, wavelet-domain, and physical structural features. The summarized contributions of this research study are as follows:

(1) An empirical study was performed to introduce a universal availability method to utilizes the built-in inertial measurement sensors (IMU) of a smartphone without any additional facilities to detect the user IO state in the wild.
(2) Since people use the smartphone in their own style and the number of activities done by a person during a day can be different for different ages and genders [27], so the effect of imbalanced data is analyzed for user IO state recognition.
(3) The sensitivity and discriminability of features were measured to provide an in-depth understanding of the dimensionality reduction effects on recognition rates of the detectors in each activity dataset.

The rest of the paper are divided as follows: the related works are described in Section 2. Then, the critical points of the proposed approach are defined in Section 3. After that, the general details of our approach methodology are explained in Section 4, followed by a description of the dataset and experimental setups in separate sections. The brief description of classification methods was prepared in Section 5. Finally, in Section 6 and Section 7, we discuss the results and offer some suggestions on the possible extensions of this work in the future in Section 8. In addition, a comparison with previous work is provided in the Discussion section.

## 2. Related Work 

The indoor/outdoor detection issue has been considered by many researchers and can be categorized into two main approaches: vision-based and sensor-based. Vision-based methods depend on the hardware used and conditional parameters and cannot be an optimal design solution for detecting the environment using a smart mobile device platform. Therefore, the focus has moved towards sensor-based approaches to find a desirable solution for mobile device platforms. Walter et al. [28] introduced a new method including gravity, ambient light, and magnetic fields, for detecting the environment. Grove [29] distinguished the environment context by measuring the number of GSM Neighbor Signal Station (GNSS) and Wi-fi access points. The results of their study showed GNSS/no measurements could be used to distinguish indoor from outdoor places. Ravindranath et al. [30] introduced the ability of GPS lock status to infer the ambient environment indirectly. However, despite the accuracy of GPS in many applications, it can drain the battery completely after approximately 6 hours of continuous usage, and it is only accurate in open space outdoors environments [22]. Due to the above GPS-based solution drawbacks, considering the low-cost integrated sensors on smart mobile devices provides some solutions for the indoor/outdoor detection problem based on these sensors including light, magnetometer, microphone, accelerometer, and gyroscope. Table 1 shows the summary of recent methods for detecting the environment. The limitations of these approaches are as follows:

- Light and sound approaches: The intensity of sound or light may differ over time, and it can be affected by various factors such as the position of the phone (e.g., due to users’ carrying behavior when placed in a bag or clothes pocket).
- The strength of cell tower approaches: the absolute signal strength of the cell tower may be significantly different at various places according to the mobile devices’ models, which makes it difficult to define a unique rule/model for indoor/outdoor classification problems.
- Magnetic variance approach: The magnetometer’s readings are error-prone without accurate calibration. Furthermore, the magnetic detection is only available when the user moves around.
- Wi-Fi Signal: The Wi-Fi signal length can be influenced by the shielding effects of surrounding objects or even the human body. This attribute can bring lots of noise into the detection system and it poorly available in many outdoor areas. Therefore, Wi-Fi signals cannot be used as a general approach in user IO state detection problems.

There are a few indoor/outdoor detection studies based on user motion activity detection using smart mobile devices. For instance, SenseIO [24] was designed based on the connection between the previous state of the user with the current state to call the proper sensor. The Google human activity recognition API which is available in Android-based devices was used as a module of their multi-modal detection approach. Furthermore, the activity recognition model was used only as a filter for ambient environments rather than outdoor places. The experiments showed an 85% overall detection accuracy based on only a user’s activity while the performance of the framework was improved by using Wi-Fi and light modules. The approach suffers from propagation errors, and any fault in the current state can lead to an increase in the number of erroneous decisions. 

Here our primary motivation is developing the practical approach with high accuracy based on limited numbers of sensors which are widely available in most current smartphones. Our proposed method uses mobile users’ basic activities as the main source of data to determine the environment type considering the power consumption, universal sensor availability, and potential low-quality of data.

## 3. Design Criteria

The primary purpose of the proposed system is to develop a model to utilize the conventional and inexpensive sensors such as the accelerometers and gyroscopes embedded on user-accessible portable smart devices. In the proposed algorithm, user behavior analysis is targeted rather than using conventional external and internal detection systems such as GPS receivers, cell towers, and Wi-Fi access points. One of the main motivations of the proposed approach is designing a system that can easily be used by any application platform on different smart devices. Considering the complexity and reliability of the system, the proposed method covers the following critical design concerns:

(1) Simple, functional and inclusive coverage: the environment detection process should have fast, easy-to-use, and stand-alone capabilities. It should not depend heavily on the hardware. This method can be extended to other smart devices such as smartwatches and tablets with different operating systems without requiring the installation of new facilities.
(2) Trusted: the method should be able to determine the type of environment with high accuracy, and the system performance should not be decreased and dependent on different environmental conditions.
(3) Energy consumption: Since the method runs on a mobile platform, it should be able to manage the resources of the mobile device (such as batteries, memory, and processor) optimally during the workday, as the energy of smart mobile devices can be rapidly depleted.
(4) Instantaneous: The method should be provided as an integrated service component for higher-level applications. Therefore, the method should respond as fast as the changing of the environment. Any latency in the detection phase can reduce the mobile application’s performance.
(5) Comprehensive usability (general usability): The methodology should perform a detection task independently, i.e., without prior knowledge of the environment, additional hardware or specific user feedback to ensure the comprehensiveness of the proposed method in a wide range of high-level applications.

In this paper, we have assumed indoor and outdoor environments only and semi-indoors/outdoors are excluded due to the limitations of the public dataset. It should be considered that our works can be treated as a multi-classification problem which can be investigated is proper datasets are available.

## 4. Methodology

**Figure 1:**
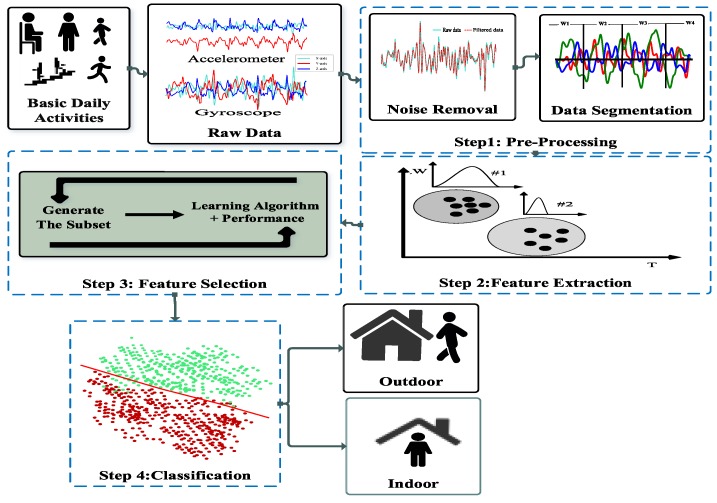
The general schema of the proposed framework.

The proposed methodology for the identification of indoor/outdoor detection environments consists of four steps including: (1) data pre-processing; (2) feature extraction; (3) feature selection and (4) classification. The data pre-processing step consists of parsing metafiles, resampling, noise reduction, and data segmentation tasks. The different temporal, frequency and wavelet domains features are extracted in the second step. In step 3, selection of the essential features is considered. Finally, in the classification step, the designing the training and testing procedures tasks and running them were examined for both Random forest and AdaBoost classifiers under different scenarios and evaluation factors. Figure 1 shows the general schema including all the steps which are described in details in the following sections: 

### 4.1. Dataset

**Figure 2:**
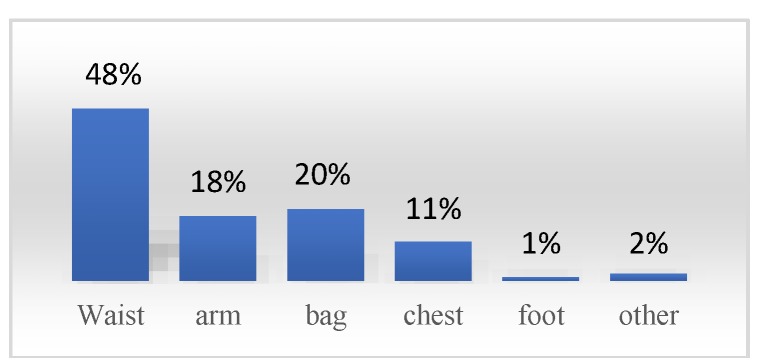
The smartphone position distribution in the HASC2016 dataset [33].

For performing trials and validation of the proposed approach, a public physical activity recognition dataset [33] has been used. The data were collected for six different activities, e.g., staying still (no activity), skip, jog, walk, climbing stairs, and going down stairs. The HASC2016 dataset [33] was collected from 320 males (23.5 ± 4.7 years; 1.76 ± 0.08 m; 76.9 ± 10.0 kg) and 120 females (22.5 ± 0.5 years; 1.58 ± 0.51 m; 59.8 ± 4.3 kg) healthy subjects during five years between 2010 to 2015 and it is available online at http://hub.hasc.jp. The data was collected separately using various smartphone brands and models, including Samsung (58%), Apple (16%), LG (9%), Sharp, Sony, HTC and other brands (1%). Moreover, the position of the smartphone on the body of the subjects is highly diverse. Figure 2 shows the distribution of phone positions during the data gathering process. Since the dataset was collected in different years, it has a wide diversity regarding floor surface types, shoe types, mounted states (free or fixed), operating systems (Android and IOS), and different types of smart devices (phones or iPads). Also, the data was collected in different sampling rates between 20 Hz and 252 Hz. In this study, all samples are resampled into 100 Hz as mentioned in [33]. Also, it was collected using different types of sensors including accelerometers, gyroscopes, magnetometers, light sensors, etc. However, in this paper, we only used the accelerometer and gyroscope data; since there were not an adequate number of other sensor samples. The raw data of each smartphone sensor is collected in a simple CVS file that is accompanied with a metafile that contains the subjects’ detailed information. Each metafile consists of the subject’s ID, gender, age group, height (cm), weight (kg), terminal position, terminal type, terminal ID, terminal mounting status (fixed or free), operating system type, sampling rate, location (indoor or outdoor), floor surface type, and footwear type.

### 4.2. Data Pre-Processing

**Figure 3:**
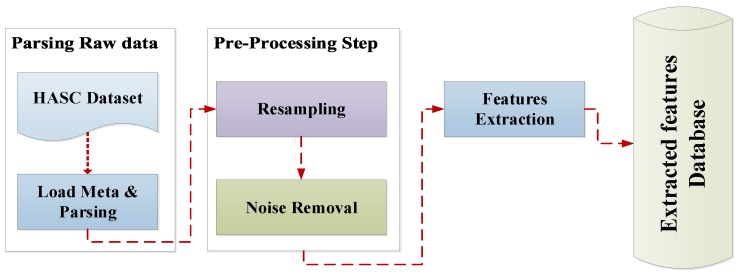
The general overview of data preprocessing.

Since a considerable amount of data was collected in the HASC2016 dataset [33], we developed an object-oriented software to convert all text data files into the database format which is more appropriate for processing a large-scale dataset. Figure 3 shows a general overview of the preprocessing steps. All files are processed and parsed in a batch process. During the preprocessing procedure, the instance features are extracted and saved in the database. Data preprocessing is divided into two steps, described below.

#### 4.2.1. Noise Removal

In general, there are different types of noise in the sensor measurements which may cause different types of uncertainty for the outputs. As a result, these noises must be removed before feeding the data as input into the model. Filtering and smoothing techniques are commonly employed to mitigate the effect of noise in obtained data. The common filtering methods for motion data include low-pass filters, high-pass filters, Kalman filters, weighted moving average (WMA), moving average filters, and smoothing algorithms [34,35]. Since the input dataset for the proposed approach is the smartphone users’ daily activities detected through their motion sensors, the 3rd order low-pass Butterworth filter with a cutoff frequency equal to 20 Hz and a median filter (N = 3) were used on each accelerometer and gyroscope axis, in way described in [36]. The cutoff frequency is set to 20 Hz because this rate is sufficient to capture human body motion since only one percent of its energy is above 15 Hz [37]. Furthermore, to increase the accuracy and reduce the uncertainty, the first two seconds and last five seconds for each sensor data file have been deleted to skip the initialization and termination behavior of data [33]. 

#### 4.2.2. Data Segmentation

Long-term data streams cannot be used directly in the form of training or testing data for most machine learning methods, so this continuous data needs to be divided into many short ones. The time-window slide segmentation method is an ordinary way of converting long-term signals into short-term ones. One possible reason can be the low-cost computational nature of this method compared with the other segmentation methods. Since our filtered data from the denoising step is still long, we segment is into many short pieces by applying an overlapped time window. In our study, each time window is 2 s, while the overlap rate is 50%.

### 4.3. Feature Extraction

Feature extraction is a complex and critical process to find and extract proper features for desirable recognition performance. Six most common daily physical activities of users were considered in this study. According to Figure 2, the smart mobile device can be at the different positions including on the subject’s body or inside a bag which is carried by users. To cover this diversity, 216 different features have been proposed which are extracted from the time, frequency, and wavelet domains. These domains generally have been used for human activity recognition systems based on smartphone motion sensors [33,38,39]. Table 2 and Table 3 show the details of the proposed feature set, including feature labels, equations and a brief description of them. The average value of each raw data on three axes (x, y, and z) is added as the fourth dimension as the norm value of three axes [33]. Then, the segmented-base features (related to each time window slice) have been extracted including all mentioned domains and physical structures of the motion behavior. These features can be considered for further analysis. In the literature, Human Activity Recognition (HAR) systems usually use time domain features because of the low computational overhead of such features [39]. The most extracted features from motion sensor data are mean, variance, maximum, minimum, median, standard deviation, correlation, and fast Fourier transform (FFT) spectral energy of the magnitude vector (MV) [34]. Both frequency and wavelet domains are too complex and require highly intensive computational processes [39,40,41] which make them hard to use for low-end devices such as smartphone platforms. Furthermore, it is critical to identify the key features in each activity dataset to compare with the other ones. In the next session, we will explain our approaches for selecting the best features of each activity dataset.

### 4.4. Feature Selection

Since many pattern recognition methods cannot cope with high-dimensional data, feature selection techniques have become an essential requirement in many applications [42]. Feature selection aims to select well-designed features, by searching for a small subset of relevant features among the original feature vectors to have better model interpretability, including higher accuracy and lower computational cost. Due to the different searching strategies, generally feature selection methods can be divided into three methods: i.e., filter, wrapper, and embedded methods. In general, filter methods select general attributes of the data to choose features subsets without including any classifier. Besides, wrapper methods need a predefined learning algorithm and use its accuracy as the evaluation measure. For example, in [43] the support vector machine (SVM) is applied on recursive feature elimination (RFE) to find the genes most related to cancers. The embedded models run feature selection during the model construction process.

In this work, the feature extraction step results in a 216-dimensionality vector. We used the recursive feature elimination method [43] with Random Forest (RF) classifier to find the optimal ranking of the extracted features from the user’s motion data. Roughly speaking, the recursive feature elimination is a wrapper method which can select features by recursively considering smaller sets of features by training the learner model on the initial set of features, and weights. Then, the features with the absolute minimum weights are removed from the present set features. The optimum number of features with the highest accuracy would be found by running the procedure iteratively. It should be noted that 10-fold cross-validation is used for dealing with the over-fitting problem of the classification task. Also, the Feature selection process is performed by using the “scikit-learn” feature of Python. We utilize these features as optimal features for AdaBoost classifier to have a fair comparison with another classifiers’ performance. Table 4 lists the names and an optimal number of features for stay activity under various scenarios which have significantly reduced dimensions by Recursive Feature Elimination and Cross-Validated (REFCV) algorithms. Furthermore, the number of features for different scenarios due to each activity data set are shown in Table 5. The details of all scenarios and data sources are given in Table A1, Table A2, Table A3, Table A4, Table A5 and Table A6 in the Appendix to avoid excessive details and elaboration of the extensive information.

## 5. Classification

Most of the commonly used classifiers are fundamentally binary classifiers (e.g., linear support vector machines, linear discriminate analysis, nearest neighbor, random forest (RF), AdaBoost, etc.). Since the indoor-outdoor detection problem is defined as a two-class classifier problem in this study, the experimental study was performed using several binary classifiers. Based on our performance analysis, the performance of RF and AdaBoost were better than the other alternatives. RF and AdaBoost are two well-known ensemble classification methods. Their performance is robust against overfitting issues, and they have a low hyperparameters to adjust, and less-parameter tuning and robustness against noise are important practical aspects. A brief description of the selected classifiers with their parameter setting is presented as the following subsections.

### 5.1. Random Forest (RF)

The random forest (RF) [44] classifier uses a tree-type classifier method for its classification, which contains several trees expanded from a bootstrapped set of the primary training dataset. RF methods are robust against of noise or overfitting problems because their resampling does not use weighting [45]. Moreover, RF can handle high dimensionality data by increasing the number of trees, which is suitable for our feature vectors given in the previous section. RF algorithms start with several bootstrapped sets created from the original training samples in the training step, and search only through a randomly chosen subset of input samples to decide a split for each node. For classification, RF has performed a vote based on the most common class in input data for each tree, and then the classifier output is determined by a majority vote of the trees. Since the number of trees is the only elastic parameter in RF, it is adjusted to 30 in our study, to achieve lower computational overload while gaining high accuracy.

### 5.2. AdaBoost (ADA)

AdaBoost (ADA) is known to be one of the best out-of-the-box classification algorithms and has been applied to numberless machine learning problems [46]. AdaBoost initializes by choosing a series of a weak learner (e.g., decision trees) and improves learners’ accuracy iteratively by changing the weights of misclassified instances in the training dataset [47]. For example, the misclassified dataset generated by the previous learner is chosen more often than a correct one, so that new classifier’s performance can be better in the new dataset. In each iteration, AdaBoost assigns an equal weight to the dataset so that the next integration focuses on reweighting the previously misclassified dataset. The final model is obtained from the weighted sum of all weak learners. In this study, we applied Python programming to implement the AdaBoost model. Based on the experimental study on optimal parameters of AdaBoost model, the number of estimators and learning rates are set to 600 and 0.1, respectively.

## 6. Model Evaluation and Results

In general, lots of data is required to achieve high accuracy in a multi-class classification problem, especially in the feature vectors are of a high dimensionality nature. In addition, collecting data is a difficult task in multi-class classification problems, which is also seen in indoor/outdoor detection problems. On the other hand, in most circumstances, one subject’s data is not sufficient to build a robust general model. Thus, the other subjects’ data may have to be added in the set, which can increase the possibility of abnormal interference issues that can be reduced by constructing a robust general model. To overcome the mentioned problems (data collection and abnormal interference issues), we were motivated to construct a general model to investigate whether the general model can determine the environment characteristics such as indoor/outdoor decisions with an acceptable accuracy or not. The model can use an internal pre-trained classifier based on a current mobile user’s daily activities data to classify the environment type.

### 6.1. Training and Testing Procedure

To evaluate our detection approach performance, we built training and testing datasets by using all the extracted features from the raw data which are stored in the database as explained in Section 4.2. A query based on our detection problem set is executed on the database and constructs a matrix of data with their labels. The shuffled matrix is split into two subsets with 67% and 33% data share which are used for the training and testing steps, respectively. We note that the class-ratio of the whole dataset may have a small difference after this division. For each trait, we compute the confusion matrix and obtain the ROC and precision-recall graphs. Furthermore, the F-score of all classes and average F-score of each class have been computed too.

### 6.2. The Experimental Results

#### 6.2.1. Stability shift by sensors data fusion

Most of the smartphones have two integrated essential motion sensors: and accelerometer and a gyroscope. The accelerometer measures three-dimensional acceleration force data while the gyroscope measures the rate of rotation data in three dimensions. Also, human activities consist of different rotation and transition movements that need to be captured for inner/outer environment detection. Therefore, selection and identification and utilization of sensors that can provide sufficient data for the aim of improving indoor/outdoor detection accuracy are crucial. In this paper, the performance of single-sensor motion data scenarios (only an accelerometer or only a gyroscope) versus a combined-sensors (accelerometer and gyroscope) scenario to detect the environment type has been described. Table 6 shows the overall F-scores of indoor/outdoor detection rate regarding a six different activity type dataset. In this table, we can compare all the observed results after running the proposed model on different input sensory datasets.

**Figure 4:**
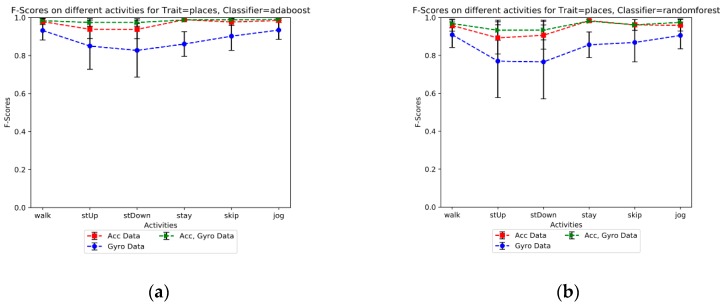
(**a**) F-score of the AdaBoost model’s performance (**b**) F-score of the Random Forest model’s performance.

**Figure 5:**
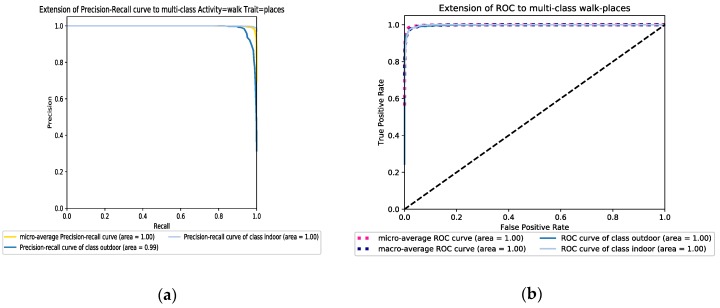
(**a**) The precision-recall performance and (**b**) the ROC graph of walk activity dataset for the Random Forest model in the combined accelerometer and gyroscope datasets scenario.

In the first scenario, we apply information resulting from an accelerator sensor (acc data) while in the second scenario, it is replaced by a gyroscope (gyro data). For the last one, we combine both datasets gathered from both sensors (acc & gyro data). Both RF and AdaBoost approaches have been applied to the three scenarios to separately expand and compare the results. The obtained results indicate that the third scenario based on data fusion of two sensors gets much better results compared to the cases where just one sensor is involved in the feature learning and classification process. Also, the results show more improvement for indoor detection accuracy compared to the outdoor detection results. For example, for RF and the stairs down activity, the outdoor performance detection rate for the three scenarios are 82.9%, 59.5%, and 87.4%, respectively compared to 97.9%, 96.2% and 98.5% for the indoor detection rates. Furthermore, a comparison between the RF and AdaBoost classifiers points out that AdaBoost has better performance than the RF one and it can achieve more than 98% accuracy for most of the activities in both indoor and outdoor detections. Besides, the performance of the AdaBoost classifier for different activities is more robust. Furthermore, the results yielded by accelerometer data show better performance than the gyroscope data for both classifiers. However, the performance of AdaBoost is acceptable enough to compare with the performance of RF for the climbing stairs and steps down activities. Figure 4a,b show the overall F-score of detectors based on the different activities. As a result, the performance of classifiers was reduced 4 % in the climbing and going down stairs activities compared with the other activities such as skip and walk. Figure 5a,b show the precision-recall and ROC graphs of walking activity under a combed accelerometer and gyroscope data scenario for both learner models. The rest of the other precision-recall and ROC graphs for different scenarios and datasets are available in the Supplementary Material section.

#### 6.2.2. Impact of Unbalanced Data

Data imbalance issues are common phenomena in various fields such as medical diagnosis, anomaly detection, economics, speaker recognition [48] and environmental concerns. They occur when the quantities of samples in datasets are not roughly equal. Most of the time, the learning process of a classifier leads to a proper fitting for dominant classes while it might be weak for minority classes, so one has to face imbalanced datasets. Oversampling the minority class and undersampling the majority class are two common solutions to deal with misbalanced datasets. Indeed, the public dataset HASC2016 is highly imbalanced so we must face this issue. In this work, instead of applying conventional methods such as a smoothing method, we manually balanced the dataset by carefully selecting subjects according to the following conditions:

(1) The subject must have participated in all activities.
(2) The subject must have both accelerometer and gyroscope motion sensor data.
(3) The number of subjects in each class must be equal to the number of subjects in the minority class.

**Figure 6:**
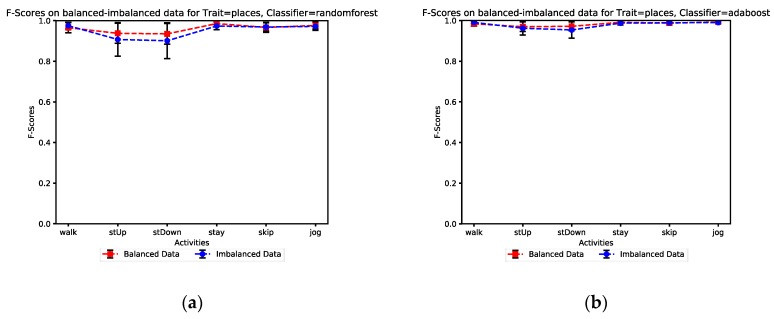
The impact of degree of unbalance on classification performance: (**a**) Random Forest classifier performance (**b**) AdaBoost classifier performance.

Figure 6a,b illustrate the comparison of the F-score of both balanced and imbalanced datasets in our model evaluation scenarios to investigate its impact on the classification performance. The results indicate that the performance is improved by considering balance issues which are well observable for the stairs up and stairs down activities. Table 7 presents the impact of a balanced dataset versus an unbalanced dataset on the proposed model. 

**Figure 7:**
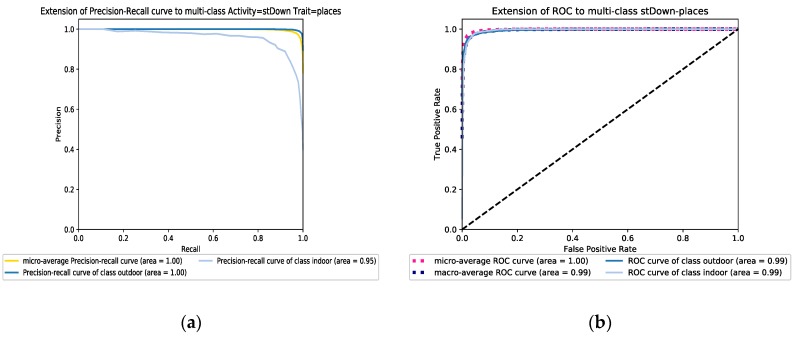
(**a**) The precision-recall graph and (**b**) the ROC graph of the Random Forest model in a balanced going down stairs dataset scenario.

The improvement is more noticeable in particular when the RF approach is deployed. Although, AdaBoost is an accuracy-oriented approach in which some dominant classes may bias its learning strategy with more contributions, the obtained results show that AdaBoost is more robust to unbalanced data compared to the RF method. Figure 7a,b illustrate the ROC and precision-recall graphs of the RF model for the balanced going stairs down dataset, respectively. The performance of both learners under different scenarios and activities dataset are provided in the Supplementary Material section.

#### 6.2.3. Sensitivity to the Feature Selection 

**Figure 8:**
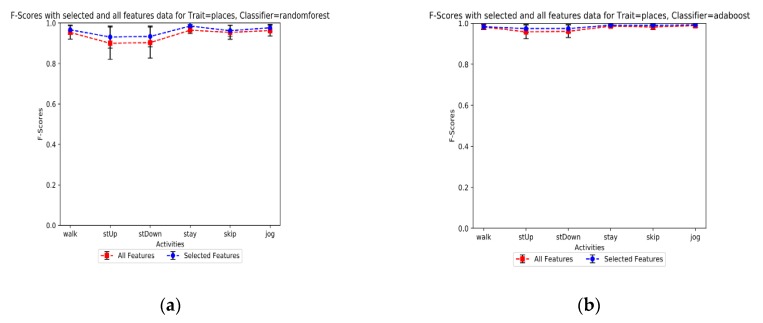
F-Score comparison for different activities. (**a**) The RF classifier model performance; (**b**) the AdaBoost classifier model performance.

**Figure 9:**
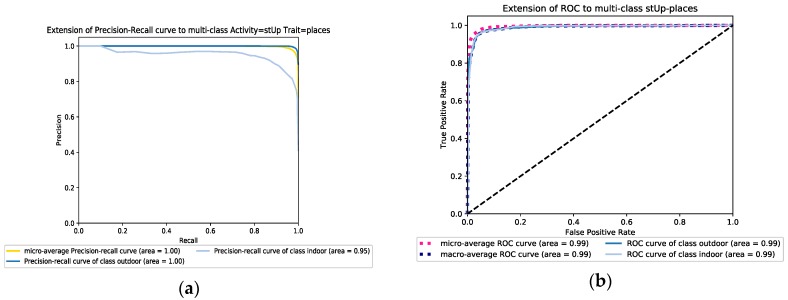
(**a**) The precision-recall graph and (**b**) the ROC graph of the RF model for the climbing stairs dataset in the selected-features scenario.

To find out the impact of selected features versus all features, some tests were performed in this study. This not only can help reduce the dimensionality of features vectors but also potentially reduce the power consumption of smartphones. For this propose, we applied different feature selection methods in our simulation, and finally, we achieved the best consistency results by the Recursive Feature Elimination with Cross-Validation (RFECV) method. The detectors ran for both “selected features” and “all features” datasets to evaluate the performance of classifiers and their behavior under various features of the dataset. The overall F-scores are then computed for both scenarios and results show AdaBoost has less sensitivity than the RF method as depicted in Figure 8a,b. The results in both feature selection and balanced dataset verify that AdaBoost can be a suitable choice for detecting the environment based on user daily activities through smartphone sensor data. Table 8 shows the evaluation results of the models in details. It can be observed that the performance of the learner model was little improved which means the selected features are strong enough to be implemented in the proposed model. In addition, the details of essential features for each activity dataset is shown from Table A1 and Table A6 in the Appendix. Furthermore, as an example, Figure 9a,b show the precision-recall and ROC graphs of the RF model for the climbing stairs dataset under the selected-features scenario, respectively. The rest of the ROC and precision-recall graphs for different scenarios and datasets are provided in the Supplementary Material section.

## 7. Discussion 

With regards to the successful outcomes of previous studies and our experimental results, we provide some perspectives to increase the smartphone indoor/outdoor detection performance through the daily activities data. Since people always reveal various movement pattern even in similar activities, we can notice that each person has his/her natural fingerprint, so without any prior knowledge about the environment, we can detect their indoor/outdoor environment completely and accurately. The fact that we got the best performance during all our experimental scenarios can verify our interpretation. Our results imply that increasing the level of sensor fusion can improve the accuracy. Furthermore, the analysis shows that accelerometer data can provide acceptable performance accuracy for environment recognition through users’ motion data.

Due to the different distribution of smartphone users and the variation in the frequency rates of smartphone’s motion sensors, a data resampling algorithm needs to be considered for indoor/outdoor detection. By utilizing a proper upsampling method, we able to increase the classifier’s accuracy. Nevertheless, the results show that the proposed model is robust enough against unbalanced data.

In contrast with other approaches, the obtained results (overall F-score) of the proposed model are shown in Table 9. The proposed model reveals better overall performance accuracy compared to other methods, for instance [18] and [24], for the indoor and outdoor detection issue. The obtained results also indicate that improvement in outdoor detection accuracy is more noticeable than with the previous approaches. Also, the proposed model can distinguish the environment type just by using the smartphone motion sensors to capture the user’s mobility trace as input data without any extra information of the situation. It should be noted that Table 9 only shows the comparison of other approaches with the proposed method in general due to the use of different sensors and experiments.

## 8. Conclusions and Future Works

Detecting the current user’s environment type and switching between them automatically is critical for many high-layer applications such as location-based services (LBS), SNAL, and healthcare monitoring. Several approaches have been developed recently to identify the inner/outer environment. Our approach is built on using the natural daily life motion of users which is recorded through smartphone motion sensors to detect the environment type without any need for extra information from other additional equipment such as mobile cell towers or Wi-Fi access points. Since the power consumption of motion sensors is seven-times less than the GPS sensor’s power consumption on the smartphone [15,16], the proposed model may address the power-hunger problem of GPS-based models. The proposed model utilizes ensemble algorithms (AdaBoost and Random Forest) on a smartphone user motion sensory dataset to recognize the different patterns of the environment profiles. Our large-scale model evaluations show that the proposed approach is capable of detecting the inner and outer environment patterns with 99% accuracy. Also, the experimental results reveal that the performance accuracy can be affected slightly by an unbalance factor. With extensive features’ analysis, the compatibility of essential selected features has been proved for each activity in the dataset. Despite the acceptable performance accuracy on the accelerometer dataset in most of the activities, the fusion of accelerometer and gyroscope data can improve the performance of the proposed model in all activities in term of F-scores. Building a comprehensive model that can cover all aspects of the problem is a challenging task. Complimentary metainformation should be merged with the current public dataset for possible improvement. For example, in real-world conditions human activity has a sequential nature. Therefore, transitional activities such as “sit to stand" or “stand to sit” can be considered as an invalid activity in our systems. The effect of transitional or other complex activities on the performance of the proposed approach can be investigated as further work. Furthermore, the stability of the patterns may be affected by slight smartphone orientation modifications, even in the same position setting, which might affect the recognition accuracy. In the future we plan to analyze a dataset of sequential human activities to improve the system stability by considering both the effects of the smartphone position and orientation.

## Figures and Tables

**Table 1:** The state-of-art for sensor-based indoor/outdoor detection approaches.

References	Sensor Types	Proposed Method	Overall Accuracy
Tempio [20], UPCASE [31]	Temperature	Environmental temperature measurements are classified using a threshold based on the user’s comfort zone and weather forecasts	----
IO Detector [18]	Light, cell, and magnetometer	The system performance was checked based on sub-detectors including light-, cell-, magnetism- and a hybrid detector	Around 85%.
Semi-supervised [15]	light, cell, and Sound	Individual modules on diverse phones are used in unfamiliar environments in three different scenarios, including cluster-then-label, self-training, and co-training	92% for unfamiliar places
Door Events [32]	Barometer	At the moment the door of a building sis opened or closed, the indoor pressure increases, and a smartphone’s barometer can measure the pressure increment.	99% for door event detection
Sound [21]	Microphone	A special chirp sound probe is propagated by a mobile device speaker and then collected back through device microphone to use as input dataset.	Roughly 95% accuracy at 46 different known places
GSM signal [22]	GSM signal of cell tower	The different GSM signal intensities of four environment types (deep indoors, semi-indoors, semi-outdoors, outdoors) are classified.	95.3%
WIFI Boost [23]	Wi-Fi	The intensity variations of Wi-Fi signals are classified into inside/outside environments or the number of access points around the devices is measured.	Around 2.5% mean error rate for familiar places.
SenseIO [24],	Accelerometer, gyroscope, light, cell, Wi-Fi	A multi-modal approach with a framework including four modules (activity recognition, light, Wi-Fi and GSM) is created.	92%

**Table 2:** The list of features extracted separately from accelerometers and gyroscopes [33,39].

Label	Description	Equation	Size
Avg_	Average of samples in each axis separately	$x¯=\frac{1}{m}\sum_{i=1}^{m}x_{i}$, where $x=\left\{a_{x},a_{y},a_{z},a_{n},g_{x},g_{y},g_{z},g_{n}\right\}$	1 × 8
SD_	The standard deviation of samples in each axis separately	$\sigma =\sqrt{\frac{1}{m}\sum_{i=1}^{m}{\left(\bar{x}-x_{i}\right)}^{2}}$, where $x=\left\{a_{x},a_{y},a_{z},a_{n},g_{x},g_{y},g_{z},g_{n}\right\}$	1 × 8
MinMax_	The difference between “Maximum Value and “Minimum Value” of samples in each axis	$\begin{array}{cc} \mathrm{MinMax}= & \mathrm{Max}{\left(x_{i}\right)}_{1\leq i\leq N}-\mathrm{Min}{\left(x_{i}\right)}_{1\leq i\leq N},\\ & \mathrm{here}\;x=\left\{a_{x},a_{y},a_{z},a_{n},g_{x},g_{y},g_{z},g_{n}\right\} \end{array}$	1 × 8
Var_	Moving variance of samples on the x-, y-, and z-axes	$\mathrm{var}=\frac{1}{N\left(N-1\right)}\left(N\sum_{i=1}^{N}x_{i}^{2}-{\left(\sum_{i=1}^{N}x_{i}\right)}^{2}\right)$ $\;\mathrm{where}\;x=\left\{a_{x},a_{y},a_{z},g_{x},g_{y},g_{z}\right\}$	1 × 6
SMA_	The simple moving average of data	${\mathrm{SMA}}_{i}=\frac{1}{N}\left(\sum_{i=1}^{N}\left|i_{x}\right|+\sum_{i=1}^{N}\left|i_{y}\right|+\sum_{i=1}^{N}\left|i_{z}\right|\right),$ where i={a,g}	1 × 2
E_	First eigenvalue of moving covariance between samples	$E_{i}={\mathrm{eig}}_{1}\left(\mathrm{cov}\left(i_{x}\left(1:N\right),i_{y}\left(1:N\right),i_{z}\left(1:N\right)\right)\right),$ where i={a,g}	1 × 2
ME_	Moving energy of sensor’s signal on each axis	$\mathrm{ME}=\frac{1}{N}\sum_{i=1}^{N}x_{i}^{2},$ $\mathrm{where}\;x=\{a_{x},a_{y},a_{z},a_{n},g_{x},g_{y},g_{z},g_{n}\}$	1 × 8
MC_	Moving correlation of sensor data between two axes	${\mathrm{MC}}_{P}^{\mathrm{xy}}=\mathrm{corr}\left(P_{x},P_{y}\right),{\mathrm{MC}}_{p}^{\mathrm{xz}}=\mathrm{corr}\left(P_{x},P_{z}\right),$ ${\mathrm{MC}}_{p}^{\mathrm{XZ}}=\mathrm{corr}\left(P_{y},P_{z}\right),\mathrm{where}\;p=\left\{a,g\right\}$	1 × 6
MMA_	The moving mean of orientation vector of sensor’s data	$\mathrm{MMA}=\frac{1}{N}\sum_{i=1}^{N}\varphi_{i},\;\mathrm{where},$ $\varphi =\frac{\arccos\left(p_{x},p_{y}\right)}{\left|p_{x}\right|.\left|p_{y}\right|},p=\left\{a,g\right\}$	1 × 2
MVA_	Moving variance of orientation vector of sensor’s data	$\mathrm{MVA}=\frac{1}{N\left(N-1\right)}\left({\left(\sum_{i=1}^{N}\varphi_{i}\right)}^{2}-\sum_{i=1}^{N}\varphi_{i}^{2}\right),$ $\mathrm{where}\;\varphi =\frac{\arccos\left(p_{x},p_{y}\right)}{\left|p_{x}\right|.\left|p_{y}\right|}\;,\;p=\left\{a,g\right\}$	1 × 2
MEA_	Moving energy of orientation vector of sensor’s data	$\mathrm{MMA}=\frac{1}{N}\sum_{i=1}^{N}\varphi_{i}^{2},\;\mathrm{where}\;\varphi =\frac{\arccos\left(p_{x},p_{y}\right)}{\left|p_{x}\right|.\left|p_{y}\right|}\;,$ p={a,g}	1 × 2
Spec.No_		The power spectrum is computed from the FFT result. From 0.5 Hz to 5 Hz (in 0.5 Hz intervals) for x, y, z, and n for both accelerometer and gyroscope	1 × 80
Wavelet_STD_index.D.No._		The standard deviation of the acceleration signal at level 2 to 5 corresponding to 0.78–18.75 Hz in three directions (i.e., AP/ML/VL) for x-, y- and z-axes	1 × 36
Wavelet_RMS_index.D.No._		Root mean square values of AP and VT acceleration signals for the x-, y-, and z-axes	1 × 24

**Table 3:** The list of features that are extracted from both accelerometer and gyroscope samples [35].

Label	Description	Equation	Size
MI_ Acc_Gyro_	The difference between the movement intensity of the accelerometer and gyroscope	${\mathrm{MI}}_{\mathrm{ag}}$ $=\sqrt{\begin{array}{c} {\left(g_{x}-a_{x}\right)}^{2}+{\left(g_{y}-a_{y}\right)}^{2}+{\left(g_{z}-a_{z}\right)}^{2} \end{array}}$	1 × 1
Var_MI_	The moving variance of sample intensity data	${\mathrm{Var}}_{\mathrm{MI}}=\frac{1}{N\left(N-1\right)}\times \left(N\sum_{i=1\;}^{N}x_{i}^{2}-{\left(\sum_{i=1}^{N}x_{i}\right)}^{2}\right),$ where $x={\mathrm{MI}}_{a},\;{\mathrm{MI}}_{g},\;{\mathrm{MI}}_{\mathrm{ag}}$	1 × 3
SMA_ Acc_Gyro_	The simple moving average of the variance between acceleration and gyroscope data	${\mathrm{SMA}}_{\mathrm{ag}}=$ $\frac{1}{N}\left(\begin{array}{c} \sum_{i=1}^{N}\left|a_{x}-g_{x}\right|+\sum_{i=1}^{N}\left|a_{y}-g_{y}\right|+\sum_{i=1}^{N}\left|a_{z}-g_{z}\right| \end{array}\right)$	1 × 1
E_ Acc_Gyro_	First eigenvalue of moving covariance of difference between acceleration and gyroscope data	$E_{\mathrm{ag}}={\mathrm{eig}}_{1}\left(\mathrm{cov}\left(a_{x}-g_{x}\;,\;a_{y}-g_{y}\;,\;a_{z}-g_{z}\right)\right)$	1 × 1
ME_ Acc_Gyro_	Moving energy of acceleration and gyroscope data	${\mathrm{ME}}_{\mathrm{ag}}=\frac{1}{N}\sum_{i=1}^{N}\begin{array}{c} {\left(x_{i}-y_{i}\right)}^{2}, \end{array}$ where $x=a_{x},a_{y},a_{z},a_{n}\;,y=g_{x},g_{y},g_{z},g_{n}$	1 × 4
MMA_ Acc_Gyro_	The moving mean of the orientation vector of the variation between acceleration and gyroscope data	${\mathrm{MMA}}_{\mathrm{ag}}=\frac{1}{N}\sum_{i=1}^{N}\varphi_{i}\;,$ where $\varphi =\frac{\arccos\left(a_{x}\times a_{y}\right)}{\left|a_{x}\right|\times \left|a_{y}\right|}-\frac{\arccos\left(g_{x}\times g_{y}\right)}{\left|g_{x}\right|\times \left|g_{y}\right|}$	1×1
MVA_ Acc_Gyro_	Moving variance of the orientation vector of the variance between acceleration and gyroscope data	${\mathrm{MVA}}_{\mathrm{ag}}$ $=\frac{1}{N\left(N-1\right)}\times \left({\left(\sum_{i=1}^{N}\varphi_{i}\right)}^{2}-\;\sum_{i=1}^{N}\varphi_{i}^{2}\;\right)\;,$ where $\varphi =\;\frac{\arccos\left(a_{x}\times a_{y}\right)}{\left|a_{x}\right|\times \left|a_{y}\right|}-\frac{\arccos\left(g_{x}\times g_{y}\right)}{\left|g_{x}\right|\times \left|g_{y}\right|}$	1 × 1
MEA_ Acc_Gyro_	Moving energy of the orientation vector of the variance between acceleration and gyroscope data	${\mathrm{MEA}}_{\mathrm{ag}}=\frac{1}{N}\sum_{i=1}^{N}\varphi_{i}^{2}\;,\mathrm{where}$ $\varphi =\frac{\arccos\left(a_{x}\times a_{y}\right)}{\left|a_{x}\right|\times \left|a_{y}\right|}-\frac{\arccos\left(g_{x}\times g_{y}\right)}{\left|g_{x}\right|\times \left|g_{y}\right|}$	1 × 1
SMAMCS_ Acc_Gyro_	Moving energy of orientation vector of sensor data	${\mathrm{SMAMCS}}_{\mathrm{ag}}=\frac{1}{N}\sum_{i=1}^{N}\left({\mathrm{MCS}}_{a,i}-{\mathrm{MCS}}_{g,i}\right)\;,$ $\mathrm{where}\;a=\left\{a_{x},a_{y},a_{z}\right\}\;\mathrm{and}\;g=\left\{g_{x},g_{y},g_{z}\right\}$	1 × 3

**Table 4:** The list of essential features for stay activity due to different scenarios.

Scenario	List of Features	Size
Only accelerometer	${\mathrm{Avg}}_{a}\left(x,y,z,n\right),{\;\mathrm{SMA}}_{a},{\;E}_{a},{\;\mathrm{ME}}_{a}\left(y\right),{\;\mathrm{ME}}_{a}\left(\mathrm{xy}\right)$	8
Only gyroscope	${\mathrm{Avg}}_{g}\left(x,y,z,n\right),{\;\mathrm{SD}}_{g}\left(z,n\right),{\;\mathrm{Minmax}}_{g}\left(x,y,z,n\right),$ $\;\mathrm{Spec}2_{g}\left(z\right),\mathrm{Spec}5_{g}\left(z,n\right),\mathrm{Spec}9_{g}\left(n\right),{\mathrm{var}}_{g}\left(y\right),$ ${\mathrm{var}}_{{\mathrm{MI}}_{g}},{\mathrm{SMA}}_{g},{\;E}_{g},{\mathrm{MC}}_{g}\left(\mathrm{xy},\mathrm{xz}\right),{\;\mathrm{ME}}_{g}\left(x,z\right),$ ${\mathrm{SMAMCS}}_{g}\left(x,y,z\right)$	25
Accelerometer and gyroscope	${\mathrm{Avg}}_{a}\left(x,y,n\right),E_{a},{\mathrm{ME}}_{a}\left(\mathrm{xy}\right),{\;\mathrm{SMAMCS}}_{g}\left(x,z\right)$	7
Balanced dataset	${\mathrm{Avg}}_{a}\left(x,y,n\right),E_{a},{\mathrm{ME}}_{a}\left(\mathrm{xy}\right),{\mathrm{Avg}}_{g}\left(z\right),$ ${\;\mathrm{SMAMCS}}_{g}\left(x,z\right)$	8
Unbalanced dataset	${\mathrm{Avg}}_{a}\left(y,n\right),{\mathrm{SMA}}_{a},{\;E}_{a},{\mathrm{ME}}_{a}\left(\mathrm{xy}\right)$	5
Selected-features	${\mathrm{Avg}}_{a}\left(x,y,n\right),E_{a},{\mathrm{ME}}_{a}\left(\mathrm{xy}\right),\;{\mathrm{ME}}_{g}\left(x,z\right)$	7

**Table 5:** The number of selected features according to different activity datasets and scenarios.

Activity	Only Gyro ^1^	Only Acc ^2^	Acc ^2^ and Gyro ^1^	Balanced Data	Unbalanced Data	Selected Features
Walk	19	25	54	43	39	42
Jog	17	36	61	40	44	59
Skip	12	34	53	36	51	51
Stay	25	8	7	8	5	7
Stairs Up	12	31	32	36	40	34
Stairs Down	18	32	48	38	24	46

^1^ Gyroscope, ^2^ Accelerometer

**Table 6:** The Overall F-score rates of user IO status due to different types of daily activity.

**Random Forest**						
**Activities**	**Only Accelerometer**		**Only Gyroscope**		**Accelerometer & Gyroscope**	
	**Outdoor**	**Indoor**	**Outdoor**	**Indoor**	**Outdoor**	**Indoor**
Walk	98.75%	92.33%	97.71%	84.95%	99.06%	94.32%
Jog	98.90%	92.86%	97.52%	83.03%	99.29%	95.55%
Skip	98.90%	92.35%	96.96%	76.01%	99.03%	93.38%
Stay	97.61%	99.07%	79.73%	92.66%	97.23%	98.92%
Stairs Up	81.56%	97.89%	61.39%	96.43%	88.79%	98.69%
Stairs Down	82.95%	97.95%	59.52%	96.17%	87.40%	98.48%
**AdaBoost**						
**Activities**	**Only Accelerometer**		**Only Gyroscope**		**Accelerometer & Gyroscope**	
	**Outdoor**	**Indoor**	**Outdoor**	**Indoor**	**Outdoor**	**Indoor**
Walk	99.38%	96.32%	98.26%	88.94%	99.51%	97.10%
Jog	99.59%	97.43%	98.37%	89.15%	99.76%	98.49%
Skip	99.36%	95.72%	97.69%	82.44%	99.72%	98.15%
Stay	98.18%	99.29%	80.51%	92.89%	98.18%	99.29%
Stairs Up	88.64%	98.67%	72.09%	97.21%	95.81%	99.49%
Stairs Down	89.06%	98.66%	69.75%	96.92%	94.86%	99.34%

**Table 7:** The impact of the unbalanced dataset on the user IO status detection.

Random Forest					AdaBoost			
Activities	Balanced Data		Unbalanced Data		Balanced Data		Unbalanced Data	
	Outdoor	Indoor	Outdoor	Indoor	Outdoor	Indoor	Outdoor	Indoor
Walk	99.03%	94.15%	99.19%	96.10%	99.48%	96.94%	99.63%	98.25%
Jog	99.40%	96.18%	99.09%	95.11%	99.77%	98.57%	99.70%	98.41%
Skip	99.15%	94.26%	98.99%	94.78%	99.76%	98.40%	99.62%	98.12%
Stay	98.11%	99.26%	96.28%	99.35%	98.79%	99.53%	97.55%	99.57%
Stairs up	88.04%	98.59%	83.18%	99.03%	94.27%	99.30%	93.13%	99.58%
Stairs down	87.33%	98.47%	81.00%	98.87%	94.96%	99.35%	91.85%	99.48%

**Table 8:** The sensitivity of detectors to the size of features and activity.

Random Forest					AdaBoost			
Activities	Selected Features		All Features		Selected Features		All Features	
	Outdoor	Indoor	Outdoor	Indoor	Outdoor	Indoor	Outdoor	Indoor
Walk	99.05%	94.24%	98.79%	92.61%	99.55%	97.34%	99.43%	96.62%
Jog	99.32%	95.69%	99.08%	94.05%	99.76%	98.49%	99.74%	98.40%
Skip	99.05%	93.46%	98.89%	92.40%	99.77%	98.49%	99.55%	97.02%
Stay	97.87%	99.17%	94.62%	97.96%	98.68%	99.48%	98.07%	99.25%
Stairs up	88.12%	98.59%	82.98%	98.09%	95.17%	99.41%	92.17%	99.07%
Stairs down	88.03%	98.52%	83.02%	98.01%	95.30%	99.39%	93.55%	99.19%

**Table 9:** The overall accuracy comparison.

	IODetector [18]		SenseIO [24]		Proposed (Random Forest)		Proposed (AdaBoost)	
					Acc & Gyro		Acc & Gyro	
	Outdoor	Indoor	Outdoor	Indoor	Outdoor	Indoor	Outdoor	Indoor
Overall Accuracy	88%	90%	91.9%	94.4%	92.94%	98.27%	99.03%	99.23%

## Supplementary Materials

The results of ROC and precision-recall graphs were provided under different scenarios and activities dataset for both the Random Forest and AdaBoost classification models, which are available online at https://1drv.ms/u/s!Ajs0rvRrvk_vkyTAapXO3-6w9sUk.

## Appendix A

Table A1, Table A2, Table A3, Table A4, Table A5 and Table A6 list the essential features for each activity dataset when running the different scenarios to find the more accurate performance in the indoor/outdoor detection problem.

**Table A1:** The list of selected features due to the different activity datasets in the only gyroscope scenario.

Feature Name	Walk	Jog	Skip	Stay	Stairs up	Stairs down
E_ Gyro	✓			✓		
SMA_ Gyro	✓	✓	✓	✓	✓	✓
Avg_ Gyro _X				✓		
Avg_ Gyro _Y	✓			✓		✓
Avg_ Gyro _Z	✓			✓		
Avg_ Gyro _N	✓	✓		✓	✓	✓
MinMax_ Gyro _X	✓	✓	✓	✓	✓	✓
MinMax_ Gyro _Y		✓	✓	✓	✓	✓
MinMax_ Gyro _Z		✓		✓		✓
MinMax_ Gyro _N	✓			✓		✓
SD_ Gyro _X						✓
SD_ Gyro _Y						✓
SD_ Gyro _Z		✓	✓	✓	✓	✓
SD_ Gyro _N				✓		
ME_ Gyro _X	✓	✓		✓	✓	✓
ME_ Gyro _Y		✓	✓			
ME_ Gyro _Z	✓	✓		✓	✓	✓
Var_ Gyro _X	✓		✓			
Var_ Gyro _Y	✓		✓	✓		
Var_ Gyro _Z	✓					
MC_ Gyro _XY	✓	✓		✓	✓	✓
MC_ Gyro _XZ	✓	✓	✓	✓	✓	✓
MC_ Gyro _YZ	✓	✓	✓		✓	
Var_MI_Gyro				✓		
SMAMCS_ Gyro _X	✓	✓	✓	✓		✓
SMAMCS_ Gyro _Y	✓	✓	✓	✓	✓	✓
SMAMCS_ Gyro _Z	✓	✓	✓	✓	✓	✓
Spec2_ Gyro _Z				✓		
Spec3_ Gyro _Y	✓					
Spec3_ Gyro _X		✓				
Spec3_ Gyro _Z		✓				
Spec5_ Gyro _Z				✓		
Spec5_ Gyro _N				✓		
Spec9_ Gyro _N				✓		

**Table A2:** List of selected features due to the different activity datasets in the accelerometer only scenario.

Feature Name	Walk	Jog	Skip	Stay	Stairs up	Stairs down
Avg_Acc_X	✓	✓	✓	✓	✓	✓
Avg_Acc_Y	✓	✓	✓	✓	✓	✓
Avg_Acc_Z	✓	✓	✓	✓	✓	✓
Avg_Acc_N	✓	✓	✓	✓	✓	✓
SD_Acc_X	✓	✓	✓			✓
SD_Acc_Y		✓	✓			
SD_Acc_Z		✓	✓			
SD_Acc_N		✓	✓		✓	✓
MinMax_Acc_X		✓	✓			
MinMax_Acc_Y	✓	✓	✓			✓
MinMax_Acc_Z		✓	✓			
MinMax_Acc_N		✓	✓		✓	✓
Var_Acc_X	✓	✓	✓		✓	
Var_Acc_Y			✓			
Var_Acc_Z	✓	✓				✓
Var_MI_Acc		✓			✓	✓
SMA_Acc	✓		✓	✓	✓	✓
E_Acc		✓		✓	✓	✓
MC_Acc_XY					✓	✓
MC_Acc_XZ	✓					
MC_Acc_YZ		✓			✓	✓
ME_Acc_X	✓	✓	✓		✓	✓
ME_Acc_Y	✓	✓	✓	✓		✓
ME_Acc_Z	✓	✓	✓			
MMA_Acc	✓	✓	✓		✓	✓
MVA_Acc			✓		✓	✓
MEA_Acc	✓				✓	✓
ME_Acc_XY		✓	✓	✓	✓	✓
ME_Acc_XZ		✓	✓		✓	
ME_Acc_YZ	✓	✓	✓		✓	✓
SMAMCS_Acc_X	✓	✓	✓		✓	✓
SMAMCS_Acc_Y	✓	✓	✓		✓	✓
SMAMCS_Acc_Z	✓	✓	✓		✓	✓
Spec9_Acc_N						✓
Spec5_Acc_N					✓	
Spec6_Acc_N					✓	
Wavelet_STD_aD2_Acc_X		✓	✓		✓	✓
Wavelet_STD_aD2_Acc_Y	✓					
Wavelet_STD_aD2_Acc_Z					✓	✓
Wavelet_STD_aD3_Acc_Y	✓					
Wavelet_STD_dA3_Acc_X	✓	✓	✓			
Wavelet_STD_dA3_Acc_Y	✓	✓	✓			
Wavelet_STD_dA3_Acc_Z		✓	✓			✓
Wavelet_STD_dD3_Acc_X		✓	✓		✓	
Wavelet_STD_dD3_Acc_Y	✓	✓				
Wavelet_STD_dD3_Acc_Z			✓		✓	✓
Wavelet_RMS_aD2_Acc_X			✓		✓	✓
Wavelet_RMS_aD2_Acc_Z						✓
Wavelet_RMS_dA3_Acc_X		✓				
Wavelet_RMS_dA3_Acc_Z	✓	✓				✓
Wavelet_RMS_dD3_Acc_X		✓	✓		✓	
Wavelet_RMS_dD3_Acc_Z					✓	

**Table A3:** List of selected features due to the different activity datasets in the accelerometer and gyroscope scenario.

Feature Name	Walk	Jog	Skip	Stay	Stairs up	Stairs down
Avg_Acc_X	✓	✓	✓	✓	✓	✓
Avg_Acc_Y	✓	✓	✓	✓	✓	✓
Avg_Acc_Z	✓	✓	✓	✓	✓	✓
Avg_Acc_N	✓	✓	✓	✓	✓	✓
SD_Acc_X	✓	✓	✓			✓
SD_Acc_Y	✓		✓			
SD_Acc_Z	✓		✓			
SD_Acc_N	✓		✓		✓	✓
MinMax_Acc_X	✓		✓			
MinMax_Acc_Y	✓	✓	✓			✓
MinMax_Acc_Z	✓		✓			
MinMax_Acc_N	✓		✓		✓	✓
Var_Acc_X	✓	✓	✓		✓	
Var_Acc_Y			✓			
Var_Acc_Z	✓	✓				✓
Var_MI_Acc	✓				✓	✓
SMA_Acc		✓	✓	✓	✓	✓
E_Acc	✓			✓	✓	✓
MC_Acc_XY					✓	✓
MC_Acc_XZ		✓				
MC_Acc_YZ	✓				✓	✓
ME_Acc_X	✓	✓	✓		✓	✓
ME_Acc_Y	✓	✓	✓	✓		✓
ME_Acc_Z	✓	✓	✓			
MMA_Acc	✓	✓	✓		✓	✓
MVA_Acc			✓		✓	✓
MEA_Acc		✓			✓	✓
ME_Acc_XY	✓		✓	✓	✓	✓
ME_Acc_XZ	✓		✓		✓	
ME_Acc_YZ	✓	✓	✓		✓	✓
SMAMCS_Acc_X	✓	✓	✓		✓	✓
SMAMCS_Acc_Y	✓	✓	✓		✓	✓
SMAMCS_Acc_Z	✓	✓	✓		✓	✓
Spec 6_ Acc _X	✓					
Spec7_ Acc _Y		✓				
Spec9_ Acc _N		✓				
Spec4_ Gyro _X		✓				
Spec6_ Gyro _X		✓				
Spec8_ Gyro _Z						✓
Avg_ Gyro _X						
Avg_ Gyro _Y						✓
Avg_ Gyro _Z						
Avg_ Gyro _N		✓	✓		✓	✓
SD_ Gyro _X		✓	✓		✓	✓
SD_ Gyro _Y		✓	✓			
SD_ Gyro _Z			✓		✓	✓
SD_ Gyro _N						
MinMax_ Gyro _X		✓	✓			
MinMax_ Gyro _Y	✓		✓			
MinMax_ Gyro _Z						
MinMax_ Gyro _N	✓		✓			
Var_ Gyro _X		✓	✓			✓
Var_ Gyro _Y	✓	✓				
Var_ Gyro _Z			✓		✓	✓
Var_MI_ Gyro	✓	✓				
SMA_ Gyro		✓	✓		✓	✓
E_ Gyro	✓	✓	✓			
MC_ Gyro _XY		✓	✓		✓	✓
MC_ Gyro _XZ	✓	✓	✓		✓	✓
MC_ Gyro _YZ	✓					✓
ME_ Gyro _X		✓	✓		✓	✓
ME_ Gyro _Y	✓	✓	✓			
ME_ Gyro _Z	✓		✓		✓	✓
SMAMCS_ Gyro _X						✓
SMAMCS_ Gyro _Y				✓		
SMAMCS_ Gyro _Z	✓	✓	✓	✓	✓	✓
Var_MI_Acc_Gyro		✓				
SMA_Acc_Gyro		✓				
E_Acc_Gyro	✓	✓	✓			
ME_Acc_Gyro_X		✓	✓			✓
ME_Acc_Gyro_Y	✓	✓	✓			
SMAMCS_Acc_Gyro_Z	✓	✓	✓		✓	✓
Wavelet_STD_aD2_Acc_X		✓				
Wavelet_STD_aD2_Acc_Y	✓	✓				
Wavelet_STD_aD2_Acc_Z					✓	✓
Wavelet_STD_aD3_Acc_Y	✓					
Wavelet_STD_aD4_Acc_Y						✓
Wavelet_STD_aD4_Acc_Z						
Wavelet_STD_dA3_Acc_X	✓	✓	✓			
Wavelet_STD_dA3_Acc_Y	✓	✓	✓			
Wavelet_STD_dA3_Acc_Z	✓	✓				
Wavelet_STD_dD3_Acc_X	✓	✓			✓	
Wavelet_STD_dD3_Acc_Y	✓	✓				
Wavelet_STD_dD3_Acc_Z			✓			✓
Wavelet_RMS_aD2_Acc_X	✓					✓
Wavelet_RMS_aD2_Acc_Z						✓
Wavelet_RMS_dA3_Acc_X	✓	✓	✓			
Wavelet_RMS_dA3_Acc_Z		✓	✓			
Wavelet_RMS_dD3_Acc_X	✓	✓	✓	✓		
Wavelet_RMS_dD3_Acc_Z		✓		✓		✓

**Table A4:** List of selected features due to the different activity datasets in the balanced dataset scenario.

Feature Name	Walk	Jog	Skip	Stay	Stairs up	Stairs down
Avg_Acc_X	✓	✓	✓	✓	✓	✓
Avg_Acc_Y	✓	✓	✓	✓	✓	✓
Avg_Acc_Z	✓	✓	✓		✓	✓
Avg_Acc_N	✓	✓		✓	✓	✓
MinMax_Acc_X		✓	✓			
MinMax_Acc_Y	✓	✓	✓			
MinMax_Acc_Z		✓	✓			
MinMax_Acc_N	✓	✓	✓			
Var_Acc_X	✓	✓	✓			✓
Var_Acc_Y						
Var_Acc_Z		✓				
Var_MI_Acc	✓	✓			✓	✓
SMA_Acc	✓		✓		✓	✓
E_Acc		✓		✓	✓	✓
MC_Acc_XY						
MC_Acc_XZ						
MC_Acc_YZ						
ME_Acc_X	✓	✓	✓		✓	✓
ME_Acc_Y	✓	✓	✓			✓
ME_Acc_Z	✓				✓	✓
MEA_Acc					✓	✓
ME_Acc_XY	✓	✓		✓	✓	✓
ME_Acc_XZ					✓	✓
ME_Acc_YZ	✓	✓			✓	✓
SMAMCS_Acc_X	✓	✓	✓		✓	✓
SMAMCS_Acc_Y	✓	✓	✓		✓	✓
SMAMCS_Acc_Z	✓	✓	✓		✓	✓
Avg_Gyro _Y						✓
Avg_Gyro _Z				✓		
ME_Acc_Gyro_X	✓	✓				✓
ME_Acc_Gyro_Y	✓	✓				
ME_Acc_Gyro_Z	✓				✓	✓
SMAMCS_Acc_Gyro_Y	✓	✓			✓	✓
SMAMCS_Acc_Gyro_Z	✓	✓			✓	✓
Wavelet_STD_aD2_Acc_Y	✓					
Wavelet_STD_aD3_Acc_Y	✓					
Wavelet_STD_dA3_Acc_X	✓	✓	✓			
Wavelet_STD_dA3_Acc_Y	✓	✓	✓			
Wavelet_STD_dA3_Acc_Z		✓				
SD_Acc_X	✓		✓			
SD_Acc_Y			✓			
SD_Acc_Z	✓					
SD_Acc_N	✓	✓			✓	✓
Avg_ Gyro _N		✓	✓		✓	✓
SD_ Gyro _X		✓	✓		✓	✓
SD_ Gyro _Y	✓		✓			
SD_ Gyro _Z						✓
SD_ Gyro _N		✓				
MinMax_ Gyro _X			✓			
MinMax_ Gyro _Y						✓
MinMax_ Gyro _N	✓					
Var_ Gyro _X		✓	✓			
Var_ Gyro _Y						
Var_ Gyro _Z					✓	✓
Var_MI_ Gyro		✓				
SMA_ Gyro	✓	✓	✓		✓	✓
E_ Gyro	✓		✓			
MC_ Gyro _XY			✓		✓	
MC_ Gyro _XZ	✓	✓	✓		✓	✓
MC_ Gyro _YZ	✓					
ME_ Gyro _X		✓	✓		✓	✓
ME_ Gyro _Y	✓	✓	✓			
ME_ Gyro _Z			✓		✓	✓
SMAMCS_ Gyro _X				✓		
SMAMCS_ Gyro _Y			✓			
SMAMCS_ Gyro _Z	✓	✓	✓	✓	✓	✓
SMA_Acc_Gyro		✓				
E_Acc_Gyro	✓					
Wavelet_STD_dD3_Acc_X	✓	✓	✓		✓	
Wavelet_STD_dD3_Acc_Y	✓	✓				
Wavelet_STD_dD3_Acc_Z					✓	✓
Wavelet_RMS_aD2_Acc_X			✓		✓	
Wavelet_RMS_aD2_Acc_Z					✓	✓
Wavelet_RMS_aD4_Acc_Z						✓
Wavelet_RMS_dA3_Acc_X	✓	✓	✓		✓	
Wavelet_RMS_dA3_Acc_Z			✓			
Wavelet_RMS_dD3_Acc_X	✓					
Wavelet_RMS_dD3_Acc_Z						✓

**Table A5:** The list of selected features due to the different activity datasets in the unbalanced scenario.

Feature Name	Walk	Jog	Skip	Stay	Stairs up	Stairs down
Avg_Acc_X	✓	✓	✓			✓
Avg_Acc_Y	✓	✓	✓	✓	✓	✓
Avg_Acc_Z	✓	✓	✓		✓	✓
Avg_Acc_N		✓	✓	✓	✓	✓
SD_Acc_X		✓	✓		✓	
SD_Acc_Y	✓	✓	✓			
SD_Acc_Z	✓					
SD_Acc_N			✓		✓	
MinMax_Acc_X		✓	✓			
MinMax_Acc_Y	✓	✓	✓			
MinMax_Acc_Z			✓			
MinMax_Acc_N	✓	✓	✓			
MEA_Acc	✓				✓	✓
ME_Acc_XY	✓		✓		✓	
ME_Acc_XZ	✓				✓	
ME_Acc_YZ	✓	✓	✓		✓	✓
SMAMCS_Acc_X	✓	✓	✓			
SMAMCS_Acc_Y	✓	✓	✓		✓	
SMAMCS_Acc_Z	✓	✓	✓		✓	✓
Spec 3_ Acc _Y	✓					
Avg_ Gyro _X	✓					
Avg_ Gyro _Z	✓					
Avg_ Gyro _N		✓	✓		✓	✓
SD_ Gyro _X		✓	✓			
SD_ Gyro _Y		✓	✓			
SD_ Gyro _Z			✓		✓	✓
MinMax_ Gyro _X	✓	✓	✓			
MinMax_ Gyro _Y			✓			
MinMax_ Gyro _Z						
MinMax_ Gyro _N		✓	✓			
Var_ Gyro _X		✓	✓			
Var_ Gyro _Y			✓			
Var_ Gyro _Z			✓		✓	
Var_MI_ Gyro	✓	✓				
SMA_ Gyro		✓			✓	✓
E_ Gyro		✓	✓		✓	
Wavelet_RMS_aD2_Acc_X			✓		✓	✓
Wavelet_RMS_aD2_Acc_Z					✓	
Wavelet_RMS_dA3_Acc_X	✓	✓	✓			
Var_Acc_X	✓	✓	✓			✓
Var_Acc_Y		✓	✓			
Var_Acc_Z	✓					
Var_MI_Acc		✓	✓			✓
SMA_Acc	✓		✓	✓	✓	✓
E_Acc				✓	✓	✓
MC_Acc_XY				✓		
ME_Acc_X	✓	✓	✓		✓	
ME_Acc_Y	✓	✓	✓		✓	
ME_Acc_Z	✓		✓		✓	
MMA_Acc					✓	
MVA_Acc	✓				✓	
MC_ Gyro _XY			✓		✓	
MC_ Gyro _XZ	✓	✓	✓		✓	✓
MC_ Gyro _YZ	✓					
ME_ Gyro _X		✓	✓		✓	✓
ME_ Gyro _Y		✓	✓		✓	
ME_ Gyro _Z	✓				✓	✓
SMAMCS_ Gyro _X		✓	✓		✓	
SMAMCS_ Gyro _Y		✓	✓			
SMAMCS_ Gyro _Z	✓	✓	✓		✓	
SMA_Acc_Gyro			✓			
E_Acc_Gyro	✓		✓			
ME_Acc_Gyro_X		✓	✓		✓	
ME_Acc_Gyro_Y		✓	✓			
ME_Acc_Gyro_Z	✓				✓	✓
SMAMCS_Acc_Gyro_X			✓			
SMAMCS_Acc_Gyro_Y					✓	✓
SMAMCS_Acc_Gyro_Z			✓	✓	✓	✓
Wavelet_STD_aD2_Acc_Y	✓					
Wavelet_STD_dA3_Acc_X			✓	✓		
Wavelet_STD_dA3_Acc_Y	✓	✓	✓			
Wavelet_STD_dA3_Acc_Z		✓	✓			
Wavelet_STD_dD3_Acc_X	✓	✓	✓		✓	
Wavelet_STD_dD3_Acc_Y	✓	✓	✓			
Wavelet_STD_dD3_Acc_Z						✓
Wavelet_RMS_dA3_Acc_Z			✓			
Wavelet_RMS_dD3_Acc_X	✓	✓	✓		✓	
Wavelet_RMS_dD3_Acc_Z					✓	✓

**Table A6:** The list of selected features due to the different activity dataset in the feature-selected scenario.

Feature Name	Walk	Jog	Skip	Stay	Stairs up	Stairs down
Avg_Acc_X	✓	✓	✓	✓	✓	✓
Avg_Acc_Y	✓	✓	✓	✓	✓	✓
Avg_Acc_Z	✓	✓	✓		✓	✓
Avg_Acc_N	✓	✓	✓	✓	✓	✓
SD_Acc_X	✓	✓	✓			✓
SD_Acc_Y		✓				
SD_Acc_Z	✓		✓			
SD_Acc_N		✓	✓		✓	✓
MinMax_Acc_X		✓	✓			
MinMax_Acc_Y	✓	✓	✓			
MinMax_Acc_Z		✓	✓			
MinMax_Acc_N	✓	✓	✓			
Var_Acc_X	✓	✓	✓			
Var_Acc_Y		✓	✓			
Var_Acc_Z	✓					
Var_MI_Acc		✓	✓			✓
SMA_Acc	✓		✓		✓	✓
E_Acc		✓	✓	✓	✓	✓
Avg_ Gyro _N		✓	✓		✓	✓
SD_ Gyro _X		✓	✓			
SD_ Gyro _Y		✓				
SD_ Gyro _Z			✓		✓	✓
SD_Gyro_N		✓				
MinMax_ Gyro _X		✓				
MinMax_ Gyro _Y			✓			
MinMax_ Gyro _Z		✓				
MinMax_ Gyro _N	✓		✓			
Var_ Gyro _X		✓	✓		✓	✓
Var_ Gyro _Y	✓		✓			
Var_ Gyro _Z						✓
Var_MI_ Gyro		✓				
SMA_ Gyro		✓	✓		✓	✓
E_ Gyro	✓	✓	✓			
MC_ Gyro _XY		✓	✓			
MC_Acc_XY				✓		
MC_Acc_YZ		✓			✓	
ME_Acc_X	✓	✓	✓		✓	✓
ME_Acc_Y	✓	✓	✓			✓
ME_Acc_Z	✓				✓	✓
MMA_Acc	✓	✓			✓	✓
MVA_Acc					✓	✓
MEA_Acc	✓	✓			✓	✓
ME_Acc_XY	✓	✓			✓	✓
ME_Acc_XZ	✓	✓	✓			✓
ME_Acc_YZ	✓	✓			✓	✓
SMAMCS_Acc_X	✓	✓	✓		✓	✓
SMAMCS_Acc_Y	✓	✓	✓			✓
SMAMCS_Acc_Z	✓	✓	✓		✓	✓
Spec3_Acc_N		✓				
Spec5_Acc_Y		✓				
Spec9_Gyro_Z					✓	
Avg_ Gyro _Y						✓
SMA_Acc_Gyro		✓	✓			
E_Acc_Gyro		✓				
ME_Acc_Gyro_X	✓	✓	✓		✓	✓
ME_Acc_Gyro_Y	✓	✓	✓			
ME_Acc_Gyro_Z	✓		✓		✓	✓
SMAMCS_Acc_Gyro_Y	✓	✓	✓		✓	✓
SMAMCS_Acc_Gyro_Z	✓	✓	✓		✓	✓
Wavelet_E_Acc_Z						✓
Wavelet_STD_aD2_Acc_X					✓	✓
Wavelet_STD_aD2_Acc_Y	✓	✓				
Wavelet_STD_aD2_Acc_Z						✓
Wavelet_STD_dA3_Acc_X		✓	✓			
Wavelet_STD_dA3_Acc_Y	✓	✓	✓			
Wavelet_STD_dA3_Acc_Z		✓	✓			✓
Wavelet_STD_dD3_Acc_X	✓	✓	✓		✓	
Wavelet_STD_dD3_Acc_Y	✓		✓			
MC_ Gyro _XZ	✓	✓	✓		✓	✓
MC_ Gyro _YZ	✓					✓
ME_ Gyro _X		✓	✓		✓	✓
ME_ Gyro _Y	✓	✓	✓	✓		✓
ME_ Gyro _Z		✓	✓	✓	✓	✓
SMAMCS_ Gyro _X						✓
SMAMCS_ Gyro _Y						✓
SMAMCS_ Gyro _Z	✓	✓	✓		✓	✓
Var_MI_Acc_Gyro	✓	✓				
Wavelet_STD_dD3_Acc_Z					✓	✓
Wavelet_RMS_aD2_Acc_X		✓	✓			
Wavelet_RMS_dA3_Acc_X	✓					
Wavelet_RMS_aD4_Acc_Z						✓
Wavelet_RMS_dA3_Acc_X	✓	✓				
Wavelet_RMS_dA3_Acc_Z		✓	✓			
Wavelet_RMS_dD3_Acc_X	✓	✓				
Wavelet_RMS_dD3_Acc_Y						
Wavelet_RMS_dD3_Acc_Z					✓	✓
